# Person‐Centred Climate Despite Unmet Fundamental Care Needs: Findings From inCHARGE Using a Convergent Parallel Mixed Methods Design

**DOI:** 10.1002/nop2.70974

**Published:** 2026-10-30

**Authors:** Anna Hauffman, Lena Nyholm, Eva Jangland, Therese Avallin, Camilla Fröjd, Anna‐Karin Gunnarsson, Katarina Edfeldt

**Affiliations:** ^1^ Department of Surgical Sciences Uppsala Universitet Uppsala Sweden; ^2^ Department of Surgery Uppsala University Hospital Uppsala Sweden

## Abstract

**Aim:**

To explore person‐centred care in surgical inpatient settings by examining the person‐centred climate alongside patients' experiences of the fulfilment of fundamental care needs.

**Design:**

A convergent parallel mixed methods design.

**Methods:**

Surgical inpatients (*n* = 26) completed the Person‐centred Climate Questionnaire–Patient version and participated in structured interviews, comprising binary questions with open‐ended elaborations. Quantitative and qualitative data were analysed separately and thereafter brought together through an integrated interpretation of the findings.

**Results:**

Questionnaire scores indicated a person‐centred climate. Interview findings showed that patients described staff as kind, present, and compassionate, while also reporting several unmet fundamental care needs related to information, participation, hygiene, toileting, and symptom management. The integrated findings showed that patients reported a person‐centred climate while simultaneously describing shortcomings in the delivery of fundamental care.

**Conclusion:**

These findings highlight that global assessments of person‐centred climate may overlook deficiencies in fundamental care and underscore the importance of considering multiple dimensions when evaluating person‐centred care in clinical practice.

**Implications for the Profession and/or Patient Care:**

Assessing person‐centred care requires attention to both the care climate and the extent to which patients' fundamental care needs are met. Combining structured measures with opportunities for patients to describe their care experiences may improve the identification of unmet needs and inform efforts to strengthen person‐centred care in practice.

**Impact:**

The study illustrates that being treated with kindness and goodwill does not necessarily ensure high‐quality person‐centred care. The findings are relevant for clinicians, nurse leaders, and researchers and support the use of multi‐method approaches when evaluating person‐centred care.

**Reporting Method:**

The quantitative and qualitative content in this study is reported in accordance with the relevant STROBE guidelines.

**Patient or Public Contribution:**

The project is part of a research program developed in collaboration with the nurses and nursing leaders at the care units.

**What Does This Paper Contribute to the Wider Global Clinical Community?:**

The findings highlight the importance of combining different approaches when evaluating person‐centred care. Asking about concrete care situations and experiences may help reveal unmet fundamental care needs that might otherwise remain hidden in survey responses.

## 1 Introduction

Acute surgical settings are fast‐paced environments where patients with complex and often severe conditions require continuous monitoring and fundamental nursing care. Despite these needs, surgical inpatients frequently report physical and psychological discomfort.

(Bavin et al. 2023) and previous research shows that nursing care is frequently left undone in this population (Edfeldt et al. 2024). Nursing in surgical contexts has also been described as task‐oriented, with limited attention to the individual patient (Ball et al. 2014; Olsson et al. 2025).

Such missed nursing care (Kalisch et al. 2009) is known to negatively influence patient outcomes, including adverse events, length of stay, readmissions, and patients' experience and satisfaction (Chaboyer et al. 2021).

The Fundamentals of Care (FoC) framework (Feo et al. 2018) provides a theoretical lens for understanding these challenges by emphasizing that the integration of patients' physical and psychosocial needs is mediated through nurses' relational actions and supported by the care context (Feo et al. 2018; Kitson 2018). From this perspective, the extent to which fundamental care needs are met and the relational climate in which care is delivered can be understood as foundational to person‐centredness (McCance and McCormack 2025). A person‐centred climate, defined as an environment that supports patients as individuals and aligns care with their needs and expectations (Edvardsson et al. 2008), may therefore be considered essential for enabling the delivery of fundamental care. However, it is still unclear how patients' perceptions of person‐centred climate relate to their actual experiences of fundamental care.

Today, the importance of including the patient perspective to improve quality of care is widely recognized and patient‐reported outcome and experience measures are increasingly used (Churruca et al. 2021). Instruments that capture patient experience reflect interactions with the healthcare system whereas measures of satisfaction reflect patients' evaluation of the care received, which is naturally linked to the expectations they hold (Bavin et al. 2023). The WHO library (Larson et al. 2019) states that qualitative inquiries may be needed to validate, enhance, and explain quantitative results and to provide a deeper understanding of the caring context. When qualitative interviews are conducted, the approach can vary between structured, semi‐structured, and unstructured questioning, providing different levels of detail in the result (Pyo et al. 2023). In addition, patient‐reported data are influenced by contextual factors, which are particularly relevant in inpatient settings where patients may feel dependent on staff and therefore inclined to present themselves as ‘good patients’ (Nederhof 1985). At the same time, different approaches to collecting patient experiences may provide different insights, highlighting the importance of carefully selecting methods when evaluating and interpreting patient‐reported measures (Smith and Noble 2014).

## 2 The Study

The aim of this study was to explore person‐centred care in surgical inpatient settings by examining person‐centred climate alongside patients' experiences of the fulfilment of fundamental care needs. The study is a part of the inCHARGE programme (Hauffman et al. 2024) at Uppsala University, a research programme with an interdisciplinary focus, integrating nursing science and medical humanities with the overall aim to design innovations to improve patient care.

## 3 Methods

This study uses a convergent parallel mixed methods design (Creswell and Plano Clark 2017). Quantitative and qualitative data were analysed separately and thereafter brought together through an integrated interpretation of the findings.

### 3.1 Recruitment and Setting

The data collection was undertaken at three surgical care units in a university hospital. The care units cater for patients due to surgical ailments, and admission can be both acute and planned. Patients are primarily offered single rooms with access to a hospital bed, bedside table, lockable wardrobe, armchair, TV and a private bathroom. Each room has a notice board where the responsible staff for the day writes their names, the patients daily planning and goals (e.g., sitting up in the armchair, taking a shower or a walk). The core staff caring for patients consisted of a registered nurse (RN) and a nursing assistant (NA) responsible for 5–12 patients at a time. Other professions, such as physicians, conducted daily ward rounds, while physiotherapists visited patients based on assessed needs. Cleaning at the units was carried out by an external service provider. The care‐units were short‐staffed by about 20% of the number of RNs needed (in 2022). The recruitment took place during 3 weeks in 2022, and patients were asked to participate consecutively from the occupancy list. Patients cared for at the units were eligible if aged 18 years or older and admitted on the units for > 24 h. The exclusion criteria were cognitive impairment, infectious disease, Karnofsky performance status < 20, or inability to speak and understand Swedish. The most common criteria for exclusion were cognitive impairment. A total of 40 patients were approached and received written and verbal information about the study, 14 declined to participate, most commonly due to decreased energy or physical symptoms. Data collection continued until no substantial new information emerged in the interview data, resulting in a final sample of 26 patients.

### 3.2 Data Collection and Analysis

#### 3.2.1 Person‐Centred Climate

PCC was assessed using the validated Swedish version of the person‐centred climate questionnaire–patient version (PCQ‐P) (Edvardsson et al. 2008), used with permission. Although originally developed for nursing home settings, the PCQ‐P has also been tested and recommended for use in sub‐acute and acute care environments. The instrument comprises 17 items, rated on a six‐point Likert scale, from 1 = ‘disagree completely’ to 6 = ‘agree completely,’ yielding a total score between 17 and 102 (Rantala et al. 2018). The PCQ‐P measures person‐centred climate from 3 factors: climate of safety, climate of everydayness, and climate of hospitality. A climate of safety (Items 1–10) is characterized by competent and responsive staff and a clean, well‐organized environment with private and shared spaces. A climate of everydayness (Items 11–14) is characterized by everyday elements providing a sense of normalcy, a distraction from illness and connection to the outside world. The factor of hospitality (Items 15–17) refers to an environment where people feel welcome and well cared for, characterized by being expected, seen and acknowledged (Edvardsson et al. 2008). Data from the PCQ‐P was handled on both total and subscale levels. The questionnaire was administrated to patients prior to the structured interview, together with a brief study‐specific questionnaire including sociodemographic information (presented in the result as Table 1).

**TABLE 1:** Patient characteristics^a^ (*n* = 26).

Gender *n* (%)	
Male	14 (54.0)
Female	12 (46.0)
Age	
Mean (SD)	63 (16.0)
Min‐max (Range)	23–86 (63.0)
Relationship status *n* (%)	
Single/living alone	7 (27.0)
Married/cohabitating	19 (73.0)
Reason for admission *n* (%)	
Acute surgery	6 (23.0)
Colorectal surgery	3 (11.5)
Endocrine surgery	1 (4.0)
Liver/bile surgery	4 (15.0)
Vascular surgery	3 (12.0)
Transplantation surgery	5 (19.0)
Oesophagus/ventricle surgery	2 (8.0)
Prefer not to disclose	2 (8.0)
Days at current ward *n* (%)	
1–3 days	12 (46.0)
4–8 days	8 (31.0)
9–20	3 (11.5)
> 20	3 (11.5)
Previously admitted to hospital during last year *n* (%)	19 (73.0)
Born in sweden *n* (%)	24 (92.0)
Born in another country *n* (%)	2 (8.0)
Working status *n* (%)	
Employed	10 (38.0)
Sick leave	3 (12.0)
Retired < 65 years	1 (4.0)
Retired > 65 years	12 (46.0)
Level of education *n* (%)	
Primary school	4 (15.0)
Secondary school	8 (31.0)
University < 3 years	6 (23.0)
University > 3 years	8 (31.0)

^a^ Self‐reported data retrieved by study specific questionnaire.

#### 3.2.2 Fundamental Care Needs

To capture patients' experiences of how fundamental care needs were met during their time as inpatients, structured interviews were conducted, encouraging participants to elaborate on their responses. The interview guide (Data S1) was based on the Fundamentals of Care (FoC) framework (Feo et al. 2018). The Swedish version of the framework (Muntlin and Jangland 2023) guided the development of the interview questions. The guide covered the *context of care* (in this case, the patients' immediate care environment) and the integration of care (psychosocial care needs, relational actions and physical care needs). The interview guide was pilot‐tested with members of the research team to assess the structure, resulting in minor adjustments. Using a structured approach, questions regarding the context of care, psychosocial care needs and relational actions began with binary, yes/no items followed by prompts encouraging participants to elaborate on their experiences. Such binary, or ‘forced‐choice’ questions have been recommended over Likert‐type scales to reduce social desirability bias (Brown and Maydeu‐Olivares 2011). For example: ‘Have you received sufficient information to be able to participate in your own care?’ (YES/NO) ‘Would you like to describe that further?’ The binary responses were meticulously documented in the interview guide together with field notes including patients' statements or direct quotes. Questions regarding physical care needs began by asking whether the patient required support with specific activities (e.g., eating, toileting). For each item the patients rated its importance for their wellbeing using a 5‐grade response scale ranging from ‘Completely unimportant’ to ‘Very important.’ Patients were also asked whether they had necessary support or aids in connection to each area and how they perceived the fulfilment. For example: (a) Have you experienced receiving the support/having the aids you need to manage your personal hygiene, and (b) Have you experienced being able to perform your personal hygiene in the way you desire?

Interviews were conducted during the participants' hospital stay in a conducive environment (single room) ensuring minimal disturbance. No audio recordings were made during the interviews. Instead, detailed field notes were taken and key quotations were noted verbatim. Field notes were documented during and immediately after each interview to capture participants' responses in as much detail as possible. When participants provided particularly illustrative statements, the interviewers occasionally paused to document these quotations as close to verbatim as possible. In the analysis, all short responses and ratings were compiled in a summary table. The field notes were analysed using content analysis (Graneheim and Lundman 2004) by the first author (AH) applying a low level of interpretation to stay close to the data. The analysis was inductive and conducted within the domains (context of care, psychosocial care needs, relational actions and physical care needs) corresponding to the interview guide. Thus, categories were generated from the data rather than predetermined (Graneheim and Lundman 2004). The field notes were read repeatedly and coded. Codes with shared content were grouped into categories. Binary responses were also reviewed alongside participants' elaborations to examine how the responses were expanded or nuanced in the subsequent narratives. Detailed item‐level responses are presented in (Data S2). The preliminary results and interpretations were reviewed and discussed within the author group, which led to the integration of some categories.

#### 3.2.3 Integrated Interpretation of Findings

Quantitative and qualitative data were initially analysed separately and then brought together through an integrated interpretation of the findings. During this process patients' perceptions of the person‐centred climate were discussed within the research team in relation to patients' experiences of having their fundamental care needs met, with particular attention to similarities and discrepancies.

### 3.3 Ethical Considerations

The data collection has been approved by the Swedish Ethical Review Authority (Dnr 2022–01557‐01) and is registered in a public study register (Public360 Dnr 2023–00042). Participation was voluntary and all participants provided written informed consent, in accordance with the Declaration of Helsinki (World Medical Association 2013). Given that the interviews were conducted during an ongoing hospital stay, particular attention was paid to ensuring that participation was voluntary and to clarifying that participation and responses would not influence the care received. Participants were further informed that their responses would be treated confidentially, that none of the interviewers worked in the care unit and that the purpose of the questions was to understand and improve care. The interviewers (AH, KE, LN) are all experienced RNs, nursing researchers and none had any prior relationship with the participants.

## 4 Results

Background data are presented descriptively. Results from the PCQ‐P are reported as descriptive statistics (total and subscales). Findings from the structured interviews are presented within four domains following the interview guide: context of care, psychosocial care needs, relational actions, and physical care needs. Quantitative interview findings are presented descriptively, with a particular focus on areas where patients reported unmet fundamental care needs. Detailed item‐level responses are presented in the (Data S2). Participants' physical care needs are presented descriptively. Qualitative findings are presented through the categories generated from the content analysis. The results conclude with an integrated interpretation of the findings.

### 4.1 Background Data

The sample was evenly distributed between men (54%) and women (46%); most were born in Sweden (92%) and lived together with someone else (73%). Half of the participants (50%) were retired. Around half had been in the care unit for more than 3 days; the most common reasons for the care episode were acute surgery or transplantation (Table 1).

### 4.2 Person‐Centred Climate

Patients (*n* = 26) reported the climate as person‐centred with a mean total PCQ‐P score of 86.7 (SD: 12.7), corresponding to 85% of the maximum possible score. The median score was 90.5 (IQR: 81.3–93.8), and scores ranged from 40 to 102. Among the subcategories, ‘Safety’ was rated highest with a mean score of 54.7 (91% of the maximum score of 60), followed by ‘Hospitality’ with a mean score of 14.7 (82% of the maximum score of 18), while ‘Everydayness’ received the lowest rating with a mean score of 17.4 (71% of the maximum score of 24) (Figure 1).

**FIGURE 1:**
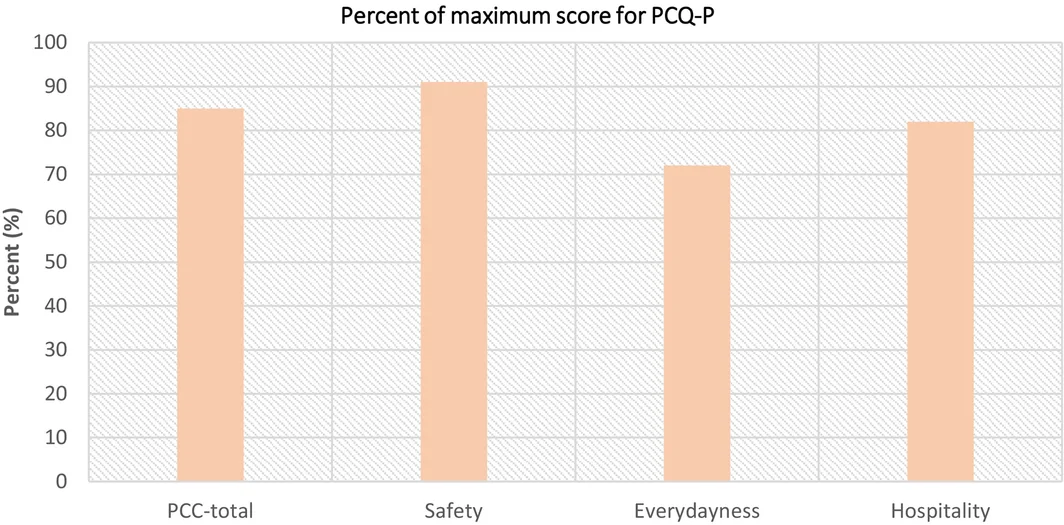
Performance of the person‐centred climate questionnaire–patient version (PCQ‐P) on total and subscale score levels (Safety, Everydayness and Hospitality) expressed as percentage of the maximum score for all patients (*n* = 26). NOTE: A climate of safety is characterized by competent and responsive staff and a clean, well‐organized environment with private and shared spaces. A climate of everydayness is characterized by everyday elements providing a sense of normalcy, a distraction from illness, and a connection to the outside world. The factor of hospitality refers to an environment where people feel welcome and well cared for, characterized by being expected, seen, and acknowledged.

### 4.3 Fundamental Care Needs‐ Quantitative Findings

The results are presented below within the four domains of the interview guide. The most frequently reported unmet fundamental care needs across all domains are presented in Figure 2.

**FIGURE 2:**
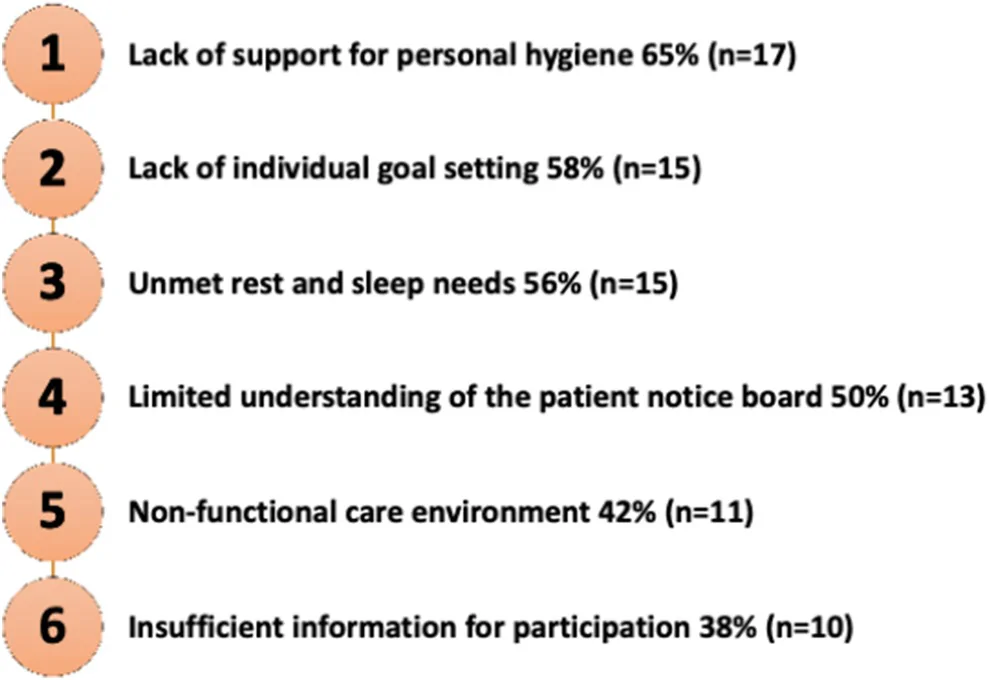
Overview of the most frequently reported unmet fundamental care needs based on negative responses (% and *n*) to structured interview questions.

#### 4.3.1 Context of Care

The highest proportion of negative responses concerned the care environment and the knowledge and usefulness of the patient's planning board. Forty‐two percent (*n* = 11) of patients did not perceive their care area as functional, and half of the patients (*n* = 13) were unfamiliar with the purpose of the daily planning board.

#### 4.3.2 Psychosocial Care Needs

When asked whether they had received sufficient information to be involved in their own care, 38% (*n* = 10) of patients reported that they had not. The majority of patients reported that their privacy and integrity had been respected. 6 patients (23%) reported experiencing stress, anxiety, and/or insecurity.

#### 4.3.3 Relational Actions

More than half of the patients (58%, *n* = 15) reported that they had not set any goals for their hospital stay together with the nursing staff. Regarding the staff's engagement in their situation, 19% (*n* = 5) stated that they did not perceive such engagement. In contrast, the highest proportion of positive responses concerned staff compassion and presence (85%, *n* = 22).

#### 4.3.4 Physical Care Needs

For physical care needs patients reported their need for support, access to support, the importance of each area for their wellbeing and the ability to handle activities as desired. The results are presented as descriptive for the areas addressed (personal hygiene, getting dressed, toileting needs, meal situation, rest and sleep, physical movement and handling of physical symptoms) (Table 2). The areas most often rated as important for wellbeing and where patients also needed support concerned the management of physical symptoms (88%, *n* = 23 needed assistance, 88%, *n* = 23 rated as important), rest and sleep (38%, *n* = 10 needed assistance, 81%, *n* = 21 rated as important), and the meal situation (46%, *n* = 10 needed assistance, 65%, *n* = 17 rated as important). In addition, patients were asked whether they had been able to manage each area as desired under the circumstances. The results showed lower results in the same areas that were rated as most important and with a greater need for support (meal situation 62%, *n* = 16, rest and sleep 65%, *n* = 17, management of physical symptoms 62%, *n* = 16). Patients were also asked if they had access to the nursing care, support or aids they needed. The pattern differed from that seen for importance and perceived ability to manage each area. The highest proportion of ‘no’ responses (65%, *n* = 17) were found for personal hygiene. In their elaborations, patients described a shortage of basic hygiene items, such as soap, toilet paper or toothbrushes. The second highest proportion of negative responses concerned rest and sleep (58%, *n* = 15). Reported problems included lack of sleep medication, discomfort (e.g., bed, pillows), and having to undergo blood sampling during the night.

**TABLE 2:** Physical care needs: Independence, lack of needed support, importance for overall wellbeing and ability to handle as desired for each area (*n* = 26).

Area	Can manage independently *n* (%)	In need of assistance *n* (%)	Reported lack of support/aids *n* (%)	Rated area as important for wellbeing^a^ *n* (%)	Managed as desired *n* (%)
Personal hygiene	17 (65)	9 (35)	17 (65)	19 (73)	20 (77)
Getting dressed	18 (70)	8 (30)	2 (8)	9 (35)	25 (96)
Toileting needs	19 (73)	7 (27)	4 (16)	18 (69)	14 (54)^b^
Meal situation	14 (54)	10 (46)	9 (35)	17 (65)	16 (62)
Rest and sleep	16 (62)	10 (38)	15 (58)	21 (81)	17 (65)
Physical movement	17 (65)	9 (35)	4 (16)	19 (73)	21 (81)
Handling of physical symptoms	3 (12)	23 (88)	7 (27)	23 (88)	16 (62)

^a^ Patients were asked to rate the importance of each area as either completely unimportant, fairly unimportant, neutral, fairly important, or very important. The percentages presented include responses fairly important and very important.

^b^ The low numbers in this area may be partly explained by patients having temporary urinary catheters and/or stomas. Please note: Percentages are based on the total sample (*n* = 26).

### 4.4 Fundamental Care Needs‐ Qualitative Findings

The content analysis generated 15 categories across the four domains, as presented in Figure 3. The categories are illustrated with direct quotations from the patients, with a total of 11 patients represented in the cited material.

**FIGURE 3:**
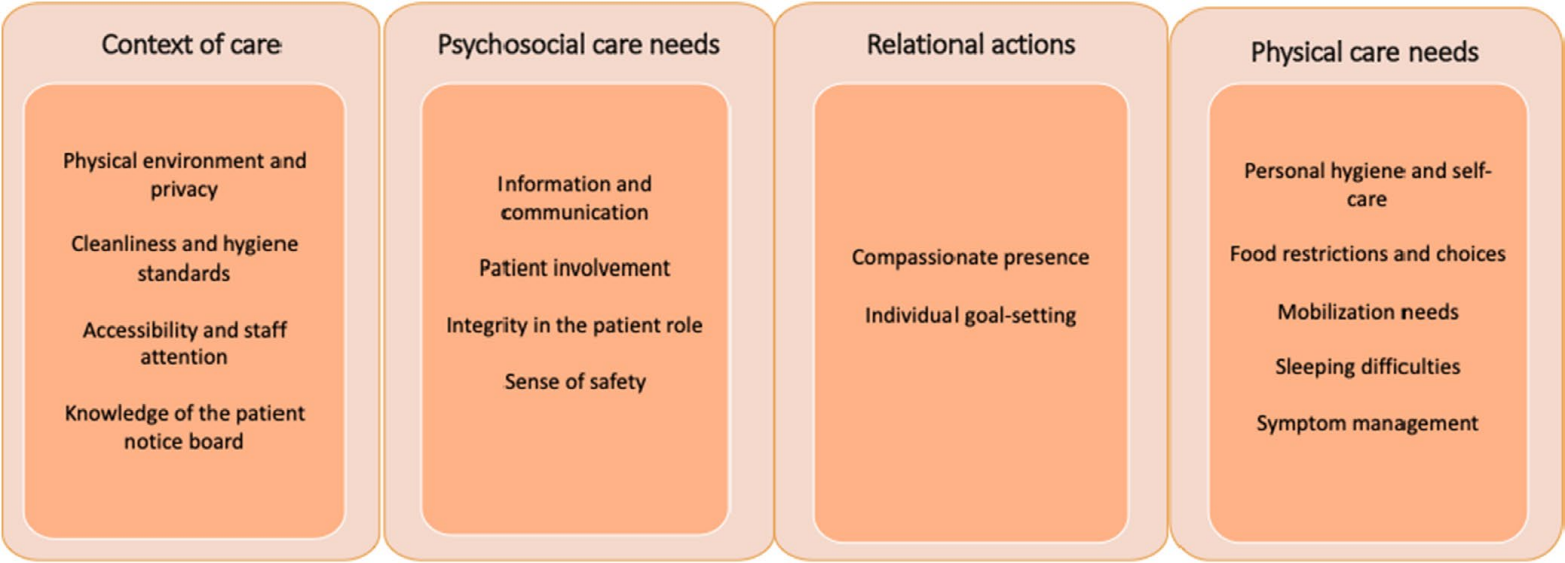
Overview of categories from the content analysis across the four domains in the interview guide.

#### 4.4.1 Context of Care

##### 4.4.1.1 Physical Environment and Privacy

Patients described the private room as a space that offered comfort and relief, particularly for those who had previously been cared for in corridors or other non‐designated areas. A room of one's own was described as a kind of sanctuary that offered a sense of autonomy, allowing patients to decide for themselves (e.g., whether to watch television, adjust the lighting, or open the window). It was perceived as a place to settle after transfers and was valued for its brightness and privacy.

I have been moved around a lot; this is the fourth room I'm in. But it feels good to have a private one. It did not feel good at all to be placed in a corridor or dayroom. Having to urinate in a bottle in the corridor as people walked by was a terrible experience.

At the same time, patients reported shortcomings in the physical environment. Missing or impractical equipment, such as uncomfortable beds or chairs (e.g., due to hygienic vinyl covers), missing hooks, or handles, made everyday activities difficult. When aids were missing, some improvised their own solutions to handle their drainage, urinary bags and intravenous stand. Issues with lighting, temperature, cramped space (due to medical devices), and lack of order further affected comfort. Feelings of insecurity also emerged when personal belongings could not be stored safely.

When I was going into surgery, I had to take off my jewellery. I locked it in the wardrobe, but I didn't know where to put the key? I couldn't take it with me, so it had to stay in the lock. It felt insecure.

##### 4.4.1.2 Cleanliness and Hygiene Standards

Having recently undergone surgery made patients particularly attentive to procedures related to daily cleaning and healthcare hygiene and some expressed concern about developing a surgical site infection. The cleaning staff were described as considerate when entering the room and the frequency of the cleaning (daily) was appreciated, while the accuracy of the cleaning e.g., stains remaining for several days, and the procedures could cause concern.

The cleaning staff cleaned my toilet and then used the same gloves on my walking frame. I think they use the same mop everywhere.

In addition, cleaning was perceived to occur mainly in common areas, while personal spaces such as the bed or bedside table were seldom cleaned. The hygienic procedures performed by healthcare staff were perceived as being carried out properly, although some inconsistencies among staff were described. Patients also expressed a sense of dependency on the staff's competence in this area. When staff followed routines such as using gloves, aprons and hand sanitizer, patients described a sense of safety and trust.

##### 4.4.1.3 Accessibility and Staff Attention

Patients reported ringing the call bell or seeking attention themselves to get contact with staff. The staff were described as friendly, but assistance was provided only upon request rather than offered proactively. Sometimes patients needed to remind staff about medication and supplies (e.g., pads, towels), and in some cases, they had to be persistent to receive help. The time for response was described as varying; the time of waiting could be especially long at night or when staff were busy. Still, patients described nursing staff as accessible and expressed sympathy and understanding of their situation.

It takes a long time, but they have too much to do—it is not due to negligence.

##### 4.4.1.4 Knowledge of Patient Notice Board

The notice boards with the purpose of showing the patient's daily plan were sometimes described as a useful tool for information e.g., names of responsible nursing staff or daily goals for physical activity. The visualization of goals could be motivating but also stressful e.g., regarding weight goals. However, patients described a common lack of knowledge and instructions for use, including inconsistency in usage.

Different staff use the board while others do not, but I have written on it myself‐ my number of walks for the day.

#### 4.4.2 Psychosocial Care Needs

##### 4.4.2.1 Information and Communication

Reported sources of misunderstandings included staff wearing face masks, speaking too quickly for patients to follow, or having a different native language which, combined with impaired hearing, contributed to communication difficulties. In such situations, patients reported strategies such as asking for clarification or, alternatively, directing their question to a different member of the healthcare team. The communication was mostly described from a perspective of receiving information or instructions. Still, patients felt that they were able to ask questions and receive responses, and the information and instructions were described as clear and reassuring. In some cases, the communication was described as mainly focusing on what needed to be done, but without an explanation. Inconsistencies in the information depending on who they talked to were also noticed.

They just tell me what to do. Eat, eat, eat. That's it.

##### 4.4.2.2 Patient Involvement

Patients' involvement in decision making was described as limited to practical matters, such as meals and dressing, and it was experienced as natural or necessary to adapt to the healthcare environment. It was described that to be involved, or sometimes even understand daily plans or routines, the questions and initiative came from the participant themselves. Being involved was also described as unexpected, or just too much to ask.

I am a patient now, so I just go along with everything.

##### 4.4.2.3 Integrity in the Patient‐Role

The patients described how their sense of integrity could be challenged during intimate care and nursing procedures. At the same time, acceptance was expressed, emphasizing that being in the hospital required them to adapt. Respectful behaviour from staff, such as knocking before entering or asking the patient to lift their shirt themselves, was described as helping to preserve a sense of dignity. Being able to maintain some control and being invited to participate was experienced as important. However, when privacy was reduced and exposure felt excessive, patients described feelings of vulnerability.

##### 4.4.2.4 Sense of Safety

The patients' experiences reflected both a sense of safety and trust in the healthcare system, as well as having support from relatives or friends. In contrast, fear and worry were described when the situation was opposite e.g., if a relative at home was unable to cope without support. Feelings of insecurity were also reported in the immediate care environment, e.g., confused fellow patients or missing personal belongings. Patients also expressed fear of being discharged too early, or concern about the consequences of the illness, including thoughts about ‘whether I will live or die’ as well as fears related to declining physical abilities.

#### 4.4.3 Relational Actions

##### 4.4.3.1 Compassionate Presence

Through everyday gestures, patients felt acknowledged and cared for as individuals rather than as tasks to be completed. Patients experienced staff as kind, attentive, and genuinely present, even within a highly structured care environment. Although the hospital setting was described as somewhat of a ‘machinery,’ staff's personal approach, such as greeting patients in the corridor, knocking before entering, asking about their wellbeing, maintaining eye contact, listening attentively, or taking time to sit down beside the patient, was appreciated as an expression of goodwill, humanity, and compassion. In contrast, when staff seemed rushed or inattentive, or when fundamental care needs such as hygiene or pain relief were not met, patients described feelings of neglect.

They come in to check that everything is going well and praise me when I'm up. They ask questions, how things are, how I'm feeling, and what I want to eat.

##### 4.4.3.2 Individual Goal Setting

Patients described limited involvement in setting individual goals for their care. Although some mentioned being informed about medical or practical objectives, such as when drains would be removed or the importance of mobilization, these goals were typically defined by staff. In addition, patients reported not being asked about their own goals or having to set them independently. The lack of information and dialogue could contribute to reduced participation in the recovery process.

I would have wished for more information about, for example, mobilization, oral care, and compression stockings. When I experienced complications, I was told that they were common. I would have liked to know beforehand to be able to prevent them.

#### 4.4.4 Physical Care Needs

##### 4.4.4.1 Personal Hygiene and Self‐Care

Patients described several challenges in maintaining personal hygiene and self‐care. Physical limitations, such as fatigue, tubes, and drains, made basic hygiene difficult. Patients also described not receiving assistance with basic hygiene such as tooth brushing or showering, or lacking essential items like soap, a mirror, or toilet paper. Feelings of neglect and resignation could emerge when hygiene needs were not prioritized or when assistance was inconsistent.

I have never felt as filthy as I do now.

Hospital garments were generally perceived as clean and functional but did not always provide sufficient coverage outside the room, contributing to feelings of exposure. Patients also reported limited independence in managing toileting needs due to catheters, stomas, or drains. Having to wait for assistance, not receiving assistance or experiencing inadequate hygiene in these situations could evoke discomfort and a desire for greater autonomy.

##### 4.4.4.2 Food Restrictions and Choices

Several challenges were described related to appetite, taste, and the environment. Patients described a lack of appetite or aversion to food due to illness, treatment, or fasting before surgery. Eating was sometimes experienced as distressing or forced. Others highlighted positive aspects, such as being able to choose their meals freely or receiving extra drinks and snacks. However, the hospital routines could interfere with meals, e.g., when examinations or ward rounds interrupted eating, making it difficult to enjoy food in peace or leaving food cold.

I have no appetite, and the chemotherapy makes everything taste awful. The staff keep insisting that I eat, but I try to explain that I just can't.

When they keep pushing, it only makes things worse.

##### 4.4.4.3 Mobilization Needs

Patients described a desire to remain active during their hospital stay but were limited by several physical aspects e.g., pain, fatigue, drains, or intravenous lines. Some patients took the initiative to get up and walk, others missed being encouraged to mobilize and would have appreciated more support or companionship. Some described the staff doing their best within the time constraints. Positive experiences were reported when staff accompanied patients on walks, which was perceived as reassuring. Patients also described loss of their usual routines and goals, e.g., daily step targets.

##### 4.4.4.4 Sleeping Difficulties

Patients experienced sleep disturbances due to environmental factors such as noise, light, uncomfortable beds, and interruptions for care procedures. Some patients were able to fall back to sleep despite these disturbances, while others described more fragmented sleep and that they had to sleep during the day. In some cases, the staff were described as doing their best to help, but patients still reported that they would have needed a sleeping pill or better pain relief to be able to rest properly. Patients also expressed a longing for the security they felt at home.

At home, I have coffee and a biscuit and watch TV… but it hasn't been like that here.

##### 4.4.4.5 Symptom Management

Patients described a wide range of symptoms, including pain, constipation, diarrhoea, nausea, fatigue, and side effects related to treatment. Experiences of the handling varied; some patients received prompt and effective symptom relief, while others described delays, uncertainty, or a lack of advice, leaving them to manage symptoms on their own. Some also described that their symptoms had not been assessed or acknowledged.

I have a dull, aching pain in my abdomen, but I haven't told anyone.

### 4.5 Integrated Interpretation of Findings

The integrated findings highlighted how the two datasets contributed related but different perspectives on person‐centred care. The clearest area of overlap concerned patients' descriptions of the care climate. High PCQ‐P scores, particularly in the dimensions of safety and hospitality, corresponded with interview accounts describing staff as kind, present, and compassionate. Participants frequently described feeling welcomed, acknowledged, and cared for by the nursing staff. Despite generally positive ratings of the care climate, several unmet fundamental care needs related to information, participation, hygiene, symptom management, and goal setting were identified across all four domains in the interview guide. Taken together, the findings identified that positive experiences of the care climate and unmet fundamental care needs were not mutually exclusive but occurred simultaneously within the same group of patients.

## 5 Discussion

The main finding of this study was that patients reported a person‐centred climate while simultaneously reporting unmet fundamental care needs. Although the PCQ‐P score fell within the upper third of the possible range, indicating a person‐centred climate, the structured interviews identified several unmet fundamental care needs. Moreover, patients described everyday goodwill and presence among staff during interviews, which could be seen as aligning with the PCQ‐P dimension of hospitality. Thus, the integrated findings show that patients may perceive the climate as person‐centred while still experiencing shortcomings in the delivery of fundamental care.

Patients described shortcomings related to information, participation, goal setting, symptom management, rest and sleep, and personal hygiene. According to the FoC framework, these aspects are interconnected and influence patients' opportunities for participation, dignity, comfort, and individualized support (Kitson 2018). Furthermore, participation, individualized support, and shared decision making are emphasized within person‐centred nursing (McCance and McCormack 2025). Difficulties in these areas may therefore have important implications for the delivery of person‐centred care in everyday practice. Moreover, the physical care needs domain showed that areas rated as most essential for wellbeing were also those in which patients had the least ability to act according to their own preferences. Barriers related to hygiene, toileting, and symptom management influenced patients' sense of dignity and comfort and are all signs of missed nursing care (Bagnasco et al. 2020; Kalisch et al. 2009). Thus, being treated with kindness and goodwill does not necessarily ensure high‐quality person‐centred care.

In line with the WHO library's view that qualitative inquiries may be needed to enhance quantitative results (Larson et al. 2019), the structured interviews provided a detailed picture of patients' care experiences. Although the PCQ‐P is a theory‐driven instrument developed to measure dimensions of person‐centred climate (Edvardsson et al. 2008), the interview guide was specifically developed to explore the fulfilment of fundamental care needs. The value of the interviews did not lie in identifying discrepancies, but in allowing patients to describe how care was experienced in everyday practice, bringing attention to both relational and practical aspects of care (Edvardsson 2015). The findings suggest that different methods capture different aspects of person‐centred care. The need to look beyond single levels of care to fully understand person‐centredness is central to the theory's origins (McCormack and McCance 2006), and the focus on fundamental care needs may be a helpful addition when seeking to identify deficiencies affecting person‐centred care in practice.

## 6 Strengths and Limitations

Although the sample size is limited, a strength of the study lies in presenting multiple forms of data from the same group of patients, enabling the identification of aspects central to person‐centred care that would remain hidden when relying on a single instrument.

Patients in poor overall health may have been less likely to participate, which could have influenced the experiences captured. In addition, participants were interviewed during an ongoing hospital stay, and their responses may have been influenced by contextual factors associated with dependency on healthcare staff. Patients may have been less willing to express criticism while still depending on the healthcare system. Thus, responses may have differed if interviews had been conducted after discharge. However, the opportunity to elaborate on their responses enabled patients to describe concerns and shortcomings that were less apparent in the initial structured responses, as illustrated in the (Data S2). Conducting the interviews during the hospital stay also ensured that experiences were recent and directly related to their ongoing care, highlighting the value of dialogue when exploring patient experiences of care (Larson et al. 2019). The interviewers were experienced nurses and were involved in developing the interview guide, which may have influenced both data collection and interpretation. To reduce this influence, the interviews followed the structured guide, and interpretations were discussed within the research team throughout the analytical process.

Furthermore, the interviews were not audio‐recorded, which can be a limitation. However, detailed field notes and verbatim quotations were documented during the interviews. Prior research suggests that carefully documented interview scripts can capture substantial detail, and that not recording can be an appropriate choice in some contexts, supporting participant comfort and openness, rather than representing a methodological compromise (Rutakumwa et al. 2019). To strengthen the trustworthiness of the data, the notes were written during and immediately after each interview, and the analysis was conducted systematically and discussed within the research team (Korstjens and Moser 2018). Although the findings are based on a specific group of surgical inpatients, the findings may be transferable to other settings where patients are similarly dependent on staff and require support with fundamental care needs.

## 7 Implications for Nursing Practice

The findings suggest that nurses should not assume that positive perceptions of a person‐centred climate necessarily reflect the fulfilment of patients' fundamental care needs. Nurses should therefore assess not only patients' overall perceptions of care but also their experiences of specific care situations. A person‐centred assessment of fundamental care needs also requires an understanding of which aspects of care are most important to the individual patient. Clinical practice may benefit from regular dialogue regarding e.g., patients' information needs, opportunities for participation, symptom burden, sleep and personal hygiene. Such assessments may support the identification of unmet needs and guide improvements in patient care. Attention should be paid to the care areas where patients experience difficulties achieving their desired level of independence, comfort or symptom control. Regular assessment of fundamental care needs may help identify care deficiencies that are less visible in broader evaluations of person‐centred care.

## 8 Recommendations for Further Research

This study contributes to methodological awareness and promotes further discussion on how person‐centred care can be measured from different perspectives. Future research is needed to explore how different approaches to evaluate person‐centred care can complement one another to a more comprehensive understanding of patients' care experiences. Studies in other patient populations and care settings are needed to examine whether the coexistence of a positive person‐centred climate and unmet fundamental care needs can be observed beyond the present study.

## 9 Conclusion

This study showed that a positive care climate and unmet fundamental care needs can coexist. Although patients frequently described staff as kind, present, and compassionate, shortcomings were identified in several areas of fundamental care. These findings suggest that understanding person‐centred care requires attention to both overall perceptions of care and patients' experiences of specific care situations. A focus on fundamental care needs may provide important insight into aspects of care that are less visible in broader assessments of the care climate.

## Author Contributions

**Anna Hauffman:** conceptualization, investigation, writing – original draft, methodology, visualization, formal analysis, data curation. **Lena Nyholm:** conceptualization, investigation, validation, writing – review and editing, formal analysis, data curation, methodology. **Eva Jangland:** conceptualization, validation, writing – review and editing, formal analysis, data curation, methodology. **Therese Avallin:** validation, writing – review and editing. **Camilla Fröjd:** validation, writing – review and editing. **Anna‐Karin Gunnarsson:** validation, writing – review and editing. **Katarina Edfeldt:** investigation, validation, writing – review and editing, data curation, formal analysis, conceptualization, methodology.

## Funding

The authors have nothing to report.

## Disclosure

Statistics: The authors confirm that the statistical aspects of this submission have been reviewed to ensure compliance with the Journal's statistical guidelines. No external statistician was consulted prior to submission, as the study is based solely on descriptive data.

## Ethics Statement

The data collection has been approved by the Swedish Ethical Review Authority (Dnr 2022–01557‐01) and is registered in a public study register (Public360 Dnr 2023–00042). Participation was voluntary and all participants provided written informed consent, in accordance with the Declaration of Helsinki (World Medical Association 2013).

## Conflicts of Interest

The authors declare no conflicts of interest.

## Supporting information


**Data S1:** nop270974‐sup‐0001‐DataS1.docx.

**Data S2:** nop270974‐sup‐0002‐DataS2.docx.

## Data Availability

The data that support the findings of this study are available on request from the corresponding author. The data are not publicly available due to privacy or ethical restrictions.

## References

[nop270974-bib-0001] Bagnasco, A. , N. Dasso , S. Rossi , et al. 2020. “Unmet Nursing Care Needs on Medical and Surgical Wards: A Scoping Review of Patients' Perspectives.” Journal of Clinical Nursing 29, no. 3–4: 347–369. 10.1111/jocn.15089. 31715037

[nop270974-bib-0002] Ball, J. E. , T. Murrells , A. M. Rafferty , E. Morrow , and P. Griffiths . 2014. “‘Care Left Undone’ During Nursing Shifts: Associations With Workload and Perceived Quality of Care.” BMJ Quality and Safety 23, no. 2: 116–125. 10.1136/bmjqs-2012-001767. PMC3913111 23898215

[nop270974-bib-0003] Bavin, E. , G. Tobiano , and B. M. Gillespie . 2023. “Patient Experience of Elective General Surgery: An Integrative Review.” Collegian 30, no. 5: 676–685. 10.1016/j.colegn.2023.08.002.

[nop270974-bib-0004] Brown, A. , and A. Maydeu‐Olivares . 2011. “Item Response Modeling of Forced‐Choice Questionnaires.” Educational and Psychological Measurement 71, no. 3: 460–502. 10.1177/0013164410375112.

[nop270974-bib-0005] Chaboyer, W. , E. Harbeck , B. O. Lee , and L. Grealish . 2021. “Missed Nursing Care: An Overview of Reviews.” Kaohsiung Journal of Medical Sciences 37, no. 2: 82–91. 10.1002/kjm2.12308. PMC11896454 33022855

[nop270974-bib-0006] Churruca, K. , C. Pomare , L. A. Ellis , et al. 2021. “Patient‐Reported Outcome Measures (PROMs): A Review of Generic and Condition‐Specific Measures and a Discussion of Trends and Issues.” Health Expectations 24, no. 4: 1015–1024. 10.1111/hex.13254. PMC8369118 33949755

[nop270974-bib-0007] Creswell, J. W. , and V. L. Plano Clark . 2017. Designing and Conducting Mixed Methods Research. 3rd ed. SAGE.

[nop270974-bib-0008] Edfeldt, K. , L. Nyholm , E. Jangland , A. K. Gunnarsson , C. Fröjd , and A. Hauffman . 2024. “Missed Nursing Care in Surgical Care: A Hazard to Patient Safety.” BMC Nursing 23, no. 1: 233. 10.1186/s12912-024-01877-1. PMC10999080 38584285

[nop270974-bib-0009] Edvardsson, D. 2015. “Notes on Person‐Centred Care: What it is and What it is Not.” Nordic Journal of Nursing Research 35, no. 2: 65–66. 10.1177/0107408315582296.

[nop270974-bib-0010] Edvardsson, D. , P. O. Sandman , and B. Rasmussen . 2008. “Swedish Language Person‐Centred Climate Questionnaire—Patient Version: Construction and Psychometric Evaluation.” Journal of Advanced Nursing 63, no. 3: 302–309. 10.1111/j.1365-2648.2008.04709.x. 18702777

[nop270974-bib-0011] Feo, R. , T. Conroy , E. Jangland , et al. 2018. “Towards a Standardised Definition for Fundamental Care: A Modified Delphi Study.” Journal of Clinical Nursing 27, no. 11–12: 2285–2299. 10.1111/jocn.14247. 29278437

[nop270974-bib-0012] Graneheim, U. H. , and B. Lundman . 2004. “Qualitative Content Analysis in Nursing Research: Concepts, Procedures and Measures to Achieve Trustworthiness.” Nurse Education Today 24, no. 2: 105–112. 10.1016/j.nedt.2003.10.001. 14769454

[nop270974-bib-0013] Hauffman, A. , E. Björk , K. Edfeldt , et al. 2024. “InCHARGE: Co‐Creating, Implementing and Evaluating Interventions to Utilize Nurses' Competence and Achieve Person‐Centred Fundamental Care—A Research Protocol Describing an Action Research Approach.” PLoS One 19, no. 7: e0304700. 10.1371/journal.pone.0304700. PMC11218940 38954688

[nop270974-bib-0014] Kalisch, B. J. , G. L. Landstrom , and A. S. Hinshaw . 2009. “Missed Nursing Care: A Concept Analysis.” Journal of Advanced Nursing 65, no. 7: 1509–1517. 10.1111/j.1365-2648.2009.05027.x. 19456994

[nop270974-bib-0015] Kitson, A. L. 2018. “The Fundamentals of Care Framework as a Point‐Of‐Care Nursing Theory.” Nursing Research 67, no. 2: 99–107. 10.1097/NNR.0000000000000271. 29489631

[nop270974-bib-0016] Korstjens, I. , and A. Moser . 2018. “Series: Practical Guidance to Qualitative Research. Part 4: Trustworthiness and Publishing.” European Journal of General Practice 24, no. 1: 120–124. 10.1080/13814788.2017.1375092. PMC8816392 29202616

[nop270974-bib-0017] Larson, E. , J. Sharma , M. A. Bohren , and Ö. Tunçalp . 2019. “When the Patient Is the Expert: Measuring Patient Experience and Satisfaction With Care.” Bulletin of the World Health Organization 97, no. 8: 563–569. 10.2471/BLT.18.225201. PMC6653815 31384074

[nop270974-bib-0018] McCance, T. , and B. McCormack . 2025. “The Person‐Centred Nursing Framework: A Mid‐Range Theory for Nursing Practice.” Journal of Research in Nursing 30, no. 1: 47–60. 10.1177/17449871241281428. PMC11907491 40093819

[nop270974-bib-0019] McCormack, B. , and T. V. McCance . 2006. “Development of a Framework for Person‐Centred Nursing.” Journal of Advanced Nursing 56, no. 5: 472–479. 10.1111/j.1365-2648.2006.04042.x. 17078823

[nop270974-bib-0020] Muntlin, Å. , and E. Jangland . 2023. “Translation and Cultural Adaptation of the Fundamentals of Care Framework: Are We There Yet?” Journal of Advanced Nursing 79, no. 3: 1107–1118. 10.1111/jan.15383. 35867344

[nop270974-bib-0021] Nederhof, A. J. 1985. “Methods of Coping With Social Desirability Bias: A Review.” European Journal of Social Psychology 15, no. 3: 263–280. 10.1002/ejsp.2420150303.

[nop270974-bib-0022] Olsson, F. , E. Jangland , and K. Edfeldt . 2025. “Experiences and Fundamental Care Needs in Patients With Small Intestinal Neuroendocrine Tumours: An Interview Study in a Surgical Context.” Journal of Advanced Nursing 81, no. 4: 2061–2071. 10.1111/jan.16441. PMC11896833 39258833

[nop270974-bib-0023] Pyo, J. , W. Lee , E. Y. Choi , S. G. Jang , and M. Ock . 2023. “Qualitative Research in Healthcare: Necessity and Characteristics.” Journal of Preventive Medicine and Public Health 56, no. 1: 12–20. 10.3961/jpmph.22.451. PMC9925284 36746418

[nop270974-bib-0024] Rantala, A. , A. Forsberg , and A. Ekwall . 2018. “Person‐Centred Climate and Psychometrical Exploration of Person‐Centredness Among Patients Not Conveyed by the Ambulance Care Service.” Scandinavian Journal of Caring Sciences 32, no. 2: 852–860. 10.1111/scs.12516. 28892188

[nop270974-bib-0025] Rutakumwa, R. , J. O. Mugisha , S. Bernays , et al. 2019. “Conducting In‐Depth Interviews With and Without Voice Recorders: A Comparative Analysis.” Qualitative Research 20, no. 5: 565–581. 10.1177/1468794119884806. PMC7444018 32903872

[nop270974-bib-0026] Smith, J. , and H. Noble . 2014. “Bias in Research.” Evidence‐Based Nursing 17, no. 4: 100–101. 10.1136/eb-2014-101946. 25097234

[nop270974-bib-0027] World Medical Association . 2013. “World Medical Association Declaration of Helsinki: Ethical Principles for Medical Research Involving Human Subjects.” JAMA 310, no. 20: 2191–2194. 10.1001/jama.2013.281053. 24141714

