# Supplementary material for: Person‐Centred Climate Despite Unmet Fundamental Care Needs: Findings From inCHARGE Using a Convergent Parallel Mixed Methods Design
Source: Nurs Open. 2026 Oct 30;13(11):e70974. doi: 10.1002/nop2.70974 (PMC13722793; doi:10.1002/nop2.70974)
Supplement: Supplementary file 1 [file NOP2-13-e70974-s001.docx]

**Supporting information 2.** Detailed item-level responses (including illustrative participant elaborations)

| Detailed item-level responses for questions concerning the patients **care environment** (n= 26) including examples of nuanced participant elaborations | | | | |
| --- | --- | --- | --- | --- |
| **Question** | **Yes n (%)** | **No n (%)** | **Yes responses with nuanced elaborations**  **n (%)** | **Yes responses* followed by nuanced elaborations** |
| 1. Do you feel that your care area (bed/bedside table/care room) is functional for your needs? | 15 (58) | 11 (42) | 1 (4) | *“I am missing a grab handle and therefore I can't get out of bed by myself”.* |
| 2. Do you feel that registered nurses and nursing assistants are accessible/can you get in touch when you want to? | 23 (88) | 3 (12) | 2 (8) | *“I have to wait for a very long time”*  *“They arrive within acceptable time”* |
| 3A. Do you feel confident that your room is being cleaned properly? | 21 (81) | 5 (20) | 2 (8) | *“I don’t think that my bedtable has been cleaned at all.”*  *“One cannot expect more”* |
| 3B. Do you feel confident that the staff are working in a hygienic manner (e.g. hand hygiene, during injections) | 23 (88) | 3 (12) | 0 | N/A |
| 4A. There is a notice board on the wall here. Do you know what it is intended for? (The intended purpose is to show patients’ daily plans, goals, and responsible staff). | 13 (50) | 13 (50) | 3 (12) | *” It says mobilization, and I had so much fun with that. It means deployment, they used to have that in the military before. I guess you’re supposed to move around? They probably write down what you do there?"*  *“They note what food is being served and such. The physician drew the stomach there”.*  *“It’s a table for their names. The name of the responsible physician is often missing.”* |
| 4B. Do you find the notice board helpful/useful? | 14 (54) | 12 (46) | 1 (4) | *“Some staff use the board, others do not.”* |
| *These patients answered "Yes" but then provided comment that added nuance to their response. The responses remain in the total for the "Yes" column*.* | | | | |

| Detailed item-level responses for questions concerning the **psychosocial care needs** (n= 26) including examples of nuanced participant elaborations | | | | |
| --- | --- | --- | --- | --- |
| **Question** | **Yes n (%)** | **No n (%)** | **Yes responses with nuanced elaborations**  **n (%)** | **Yes responses* followed by nuanced elaborations** |
| 5. Have registered nurses and nursing assistants communicated with you in a way that you could perceive and understand? | 23 (88) | 3 (12) | 3 (12) | ” *Face masks can make it difficult to hear what they are saying”.*  *“I don’t hear very well, so I have to ask again. I struggle with people who speak fast. Young women speak very quickly, so I have to ask them to repeat.”*  *“Someone from abroad came who was hard to understand, so I had to ask them to explain”.* |
| 6. Have you received sufficient information to be involved in your own care? | 16 (62) | 10 (38) | 3 (12) | *“They tell me what they think I should know”.*  *“It has become clearer over time, but I have figured out most of it myself”.*  *“I can be independent, but some things must go first like medications. But they don’t know who I really am-that’s just how it is.”* |
| 7. Have you had the privacy you need? | 23 (88) | 3 (12) | 0 | N/A |
| 8. Has your personal integrity been respected? | 25 (96) | 1 (4) | 3 (12) | *“You can’t care when you’re in the hospital-* *they must check my wound and all that. Normally, I'm pretty shy."*  *"At least, no one sighs when I need help."*  *“You can't think about it in this situation."* |
| 9. Do you feel that registered nurses and nursing assistants have respected your decisions? | 21 (81) | 5 (19) | 1 (4) | *“Aside from when I asked for pain medication, the responsiveness here is very low. They probably assume I might become a drug addict. I've received too little pain relief, even though I clearly expressed my need. It's probably the culture on the ward”.* |
| 10. Do you experience stress, anxiety and/or a sense of insecurity? | 6 (23) | 20 (77) | 2 (8) | *“I’m only worried because of my illness.”*  *“I don’t exactly stew, but I get scared when I lose my balance”* |
| *These patients answered "Yes" but then provided comment that added nuance to their response. The responses remain in the total for the "Yes" column*.* For question no 10, the opposite applies, as the comments followed a “No” response. | | | | |

| Detailed item-level responses for questions concerning the **relational actions** (n= 26) including examples of nuanced participant elaborations | | | | |
| --- | --- | --- | --- | --- |
| **Question** | **Yes n (%)** | **No n (%)** | **Yes responses with nuanced elaborations** | **Yes responses* followed by nuanced elaborations** |
| 11. Do you feel that registered nurses and nursing assistants have shown you compassion? | 22 (85) | 4 (15) | 1 (4) | “Yes, but there are very many patients here…” |
| 12. Do you feel that registered nurses and nursing assistants have been engaged in your situation? | 21 (81) | 5 (19) | 4 (15) | “They have, I suppose. I know the whole ward is overcrowded. It’s not that they ignore me”.  “Not all, but many.”  “They do a decent job. I realize how difficult it is for young people to understand how a 90-year-old functions. How much there is in one’s life. In this final period of life, a lot of change happens in a short time. They can’t get the real concept of what it’s like.”  “It obviously varies, but for the most part.” |
| 13. Do you feel that registered nurses and nursing assistants are present in their interaction with you? | 22 (85) | 4 (15) | 0 | N/A |
| 14. "Do you feel that you, together with registered nurses and nursing assistants have set goals for your stay here at the hospital?" | 11 (42) | 15 (58) | 1 (4) | "I would have wished for more information about, for example, mobilization, oral care, and compression stockings. When I experienced complications, I was told that they were common. I would have liked to know beforehand to be able to prevent them." |
| *These patients answered "Yes" but then provided comment that added nuance to their response. The responses remain in the total for the "Yes" column*.* | | | | |

| **Physical care needs** Independence, lack of needed support, importance for overall wellbeing and ability to handle as desired for each area, including examples of nuanced participant elaborations (n=26) | | | | | | | |
| --- | --- | --- | --- | --- | --- | --- | --- |
| ***Area*** | **Can manage independently**  **n (%)** | **In need of assistance**  **n (%)** | **Reported lack of needed** s**upport/aids**  **n (%)** | **Rated area as important for wellbeing** ^a).^  **n (%)** | **Managedas desired**  **n (%)** | **Yes responses with nuanced elaborations**  **n (%)** | **Yes responses* followed by nuanced elaborations** |
| *Personal hygiene* | 17 (65) | 9 (35) | 17 (65) | 19 (73) | 20 (77) | 1 (4) | “But I haven't been able to brush my teeth. I will do it when I get home”. |
| *Getting dressed* | 18 (70) | 8 (30) | 2 (8) | 9 (35) | 25 (96) | 0 | N/A |
| *Toileting needs* | 19 (73) | 7 (27) | 4 (16) | 18 (69) | 14 (54) ^b)^ | 0 | N/A |
| *Meal situation* | 14 (54) | 10 (46) | 9 (35) | 17 (65) | 16 (62) | 0 | N/A |
| *Rest and sleep* | 16 (62) | 10 (38) | 15 (58) | 21 (81) | 17 (65) | 0 | N/A |
| *Physical movement* | 17 (65) | 9 (35) | 4 (16) | 19 (73) | 21 (81) | 0 | N/A |
| *Handling of physical symptoms* | 3 (12) | 23 (88) | 7 (27) | 23 (88) | 16 (62) | 0 | N/A |
| a). Patients were asked to rate the importance of each area as either *completely unimportant, fairly unimportant, neutral, fairly important or very important.* The percentages presented include responses fairly important and very important. b). The low numbers in this area may be partly explained by patients having temporary urinary catheters and/or stomas. Please note: Percentages are based on the total sample (n=26). | | | | | | | |
