# Supplementary material for: Person‐Centred Climate Despite Unmet Fundamental Care Needs: Findings From inCHARGE Using a Convergent Parallel Mixed Methods Design
Source: Nurs Open. 2026 Oct 30;13(11):e70974. doi: 10.1002/nop2.70974 (PMC13722793; doi:10.1002/nop2.70974)
Supplement: Supplementary file 2 [file NOP2-13-e70974-s002.docx]

# Fundamentals of Care - Patient Experiences

Patient Interview Guide

*The interview guide was developed by Anna Hauffman, Katarina Edfeldt and Lena Nyholm based on the Swedish translation of the FOC framework (Muntlin, Å., & Jangland, E. (2023). Translation and cultural adaptation of the fundamentals of care framework: Are we there yet? Journal of Advanced Nursing, 79(3), 1107–1118. https://doi.org/10.1111/jan.15383).*

*This interview guide may be freely used for research or educational purposes, for any use of the guide, please cite the present article.*

## Study number: __

# Introduction

*The purpose of this interview is to give you the opportunity to describe how you experience the nursing care provided in the ward. I am a nurse and work as a researcher. I do not work in the ward. The staff working here will not know what you personally have answered. Your responses will be handled so that no unauthorised person can access them.*

*Have you read the patient information?*

*Do you have any questions about the patient information?*

*Obtain written consent – 2 copies (the patient keeps one).*

*Is it okay if we begin?*

*Please answer based on how things have been for you in the past few days. My first question concerns your immediate environment here in the ward..*

**CONTEXT OF CARE**

***The patient´s care environment***

**Question 1**

| Do you feel that your care space (bed/bedside table/room) is functional for your needs? | | |
| --- | --- | --- |
| Yes |  | If NO: Would you like to describe what you have been missing?  If YES: Would you like to describe what you appreciate about your care space? |
| No |  |  |

**Question 2**

| Do you feel that registered nurses and nursing assistants are accessible/can you get in touch when you want to? (Call button, respond when called, check in occasionally) | | |
| --- | --- | --- |
| Yes |  | If NO: Would you like to describe what you have been missing?  If YES: Would you like to describe what you have appreciated? |
| No |  |  |

**Question 3A**

| Do you feel confident that your room is being cleaned properly? | | |
| --- | --- | --- |
| Yes |  | If NO: Would you like to describe what you have been missing?  If YES: Would you like to describe what you have appreciated? |
| No |  |  |

**Question 3B**

| Do you feel confident that the staff are working in a hygienic manner? (e.g., hand hygiene, during injections, etc.) | | |
| --- | --- | --- |
| Yes |  | If NO: Would you like to describe what made you feel unsafe?  If YES: Would you like to describe what you appreciated? |
| No |  |  |

**Question 4A**

| There is a notice board on the wall here. Do you know what it is intended for? (The intended purpose is to show patients’ daily plans, goals, and responsible staff.) | | |
| --- | --- | --- |
| Yes |  |  |
| No |  |  |

**Question 4B**

| Do you find the notice board helpful/useful? | | |
| --- | --- | --- |
| Yes |  | If NO: Would you like to describe what you have been missing?  If YES: Would you like to describe how the board has been helpful to you? |
| No |  |  |

**INTEGRATION OF CARE**

***Psychosocial care needs***

**Question 5**

| Have registered nurses and nursing assistants communicated with you in a way that you could perceive and understand? (This includes verbally and in writing, hearing, level of communication, language, and avoiding overly medical terminology.) | | |
| --- | --- | --- |
| Yes |  | If NO: Would you like to describe what you feel has been missing?  If YES: Would you like to describe what you appreciated? |
| No |  |  |

**Question 6**

| Have you received sufficient information to be involved in your own care? | | |
| --- | --- | --- |
| Yes |  | If NO: Would you like to describe what you feel has been missing?  If YES: Would you like to describe what you appreciated? |
| No |  |  |

**Question 7**

| Have you had the privacy you need? (This includes the right to privacy, maintaining dignity, and ensuring that sensitive information is not discussed where others can hear.) | | |
| --- | --- | --- |
| Yes |  | If NO: Would you like to describe what you experienced?  If YES: Would you like to describe what you appreciated? |
| No |  |  |

**Question 8**

| Has your personal integrity been respected? (This is especially relevant in situations where you need assistance, such as using the toilet or personal hygiene.) | | |
| --- | --- | --- |
| Yes |  | If NO: Would you like to describe what you experienced?  If YES: Would you like to describe what you appreciated? |
| No |  |  |

**Question 9**

| Do you feel that registered nurses and nursing assistants have respected your decisions? (This includes respect for your person, your way of being, and your right to make your own choices.) | | |
| --- | --- | --- |
| Yes |  | If NO: Would you like to describe what made you feel disrespected?  If YES: Would you like to describe what you appreciated? |
| No |  |  |

**Question 10**

| Do you experience stress, anxiety, and/or a sense of insecurity? | | |
| --- | --- | --- |
| Yes |  | If YES: Would you like to describe what you have experienced? |
| No |  |  |

***Relational actions***

**Question 11**

| Do you feel that registered nurses and nursing assistants have shown you compassion? | | |
| --- | --- | --- |
| Yes |  | If NO: Would you like to describe what you experienced?  If YES: Would you like to describe what you appreciated? |
| No |  |  |

**Question 12**

| Do you feel that registered nurses and nursing assistants have been engaged in you and your situation? | | |
| --- | --- | --- |
| Yes |  | If NO: Would you like to describe what you experienced?  If YES: Would you like to describe what you appreciated? |
| No |  |  |

**Question 13**

| Do you feel that registered nurses and nursing assistants are present in their interactions with you? (e.g., have time to listen) | | |
| --- | --- | --- |
| Yes |  | If NO: Would you like to describe what you experienced?  If YES: Would you like to describe what you appreciated? |
| No |  |  |

**Question 14**

| Do you feel that you, together with registered nurses and nursing assistants, have set goals for your stay at the hospital? | | |
| --- | --- | --- |
| Yes |  | If NO: Would you like to describe what you experienced?  If YES: Would you like to describe what you appreciated? |
| No |  |  |

***Physical care needs***

**Question 15**

| **A.** Do you need nursing care/support/aids in connection with personal hygiene, e.g., brushing teeth, showering? | | |
| --- | --- | --- |
| Yes |  |  |
| No |  |  |

| **B.** Have you felt that you received the support/had the aid you needed to manage your personal hygiene? | | |
| --- | --- | --- |
| Yes |  | If NO: Would you like to describe what you have been missing?  If YES: Would you like to describe what you appreciated? |
| No |  |  |

| **C.** Considering your situation, have you felt able to manage your personal hygiene in the way you prefer? | | |
| --- | --- | --- |
| Yes |  | If NO: What do you consider the main reason for this? |
| No |  |  |

**D.** How important is this (personal hygiene) to you?

Not at all important, Somewhat unimportant, Neutral, Somewhat important, Very important

**Question 16**

| **A.** Do you need nursing care/support/aids in connection with dressing? | | |
| --- | --- | --- |
| Yes |  |  |
| No |  |  |

| **B.** Have you felt that you received the support/had the aid you needed with dressing? | | |
| --- | --- | --- |
| Yes |  | If NO: Would you like to describe what you have been missing? |
| No |  |  |

| **C.** Considering your situation, have you had the opportunity to dress in a way that feels appropriate/good for you? | | |
| --- | --- | --- |
| Yes |  | If NO: What do you consider the main reason for this? |
| No |  |  |

**D**. How important is this (your dressing) to you?

Not at all important, Somewhat unimportant, Neutral, Somewhat important, Very important

**Question 17**

| **A.** Do you need nursing care/support/aids in connection with toilet needs/CAD/stoma? | | |
| --- | --- | --- |
| Yes |  |  |
| No |  |  |

| **B.** Have you felt that you received the support/had the aid you needed for your toilet needs? | | |
| --- | --- | --- |
| Yes |  | If NO: Would you like to describe what you have been missing? |
| No |  |  |

| **C.** Considering your situation, have you had the opportunity to manage your toilet needs in a way that feels appropriate/good for you? | | |
| --- | --- | --- |
| Yes |  | If NO: What do you consider the main reason for this? |
| No |  |  |

**D**. How important is this (managing toileting needs) to you?

Not at all important, Somewhat unimportant, Neutral, Somewhat important, Very important

**Question 18**

| A. Are you currently allowed to eat and drink?   If YES: Do you need nursing care/support/aids in connection with eating and drinking? | | |
| --- | --- | --- |
| Yes |  |  |
| No |  |  |

| **B.** Have you felt that you received the support/had the aid you needed to eat and drink? | | |
| --- | --- | --- |
| Yes |  | If NO: Would you like to describe what you have been missing? |
| No |  |  |

| **C.** Considering your situation, have you had the opportunity to eat and drink in a way that feels appropriate/good for you? | | |
| --- | --- | --- |
| Yes |  | If NO: What do you consider the main reason for this? |
| No |  |  |

**D**. How important is this (eating and drinking) to you?

Not at all important, Somewhat unimportant, Neutral, Somewhat important, Very important

**Question 19**

| **A.** Do you need nursing care/support/aids in connection with resting or sleeping? | | |
| --- | --- | --- |
| Yes |  |  |
| No |  |  |

| **B.** Have you felt that you received the support/had the aid you needed for this? | | |
| --- | --- | --- |
| Yes |  | If NO: Would you like to describe what you have been missing? |
| No |  |  |

| **C.** Considering your situation, have you had the opportunity to sleep and rest in a way that feels appropriate/good for you? | | |
| --- | --- | --- |
| Yes |  | If NO: What do you consider the main reason for this? |
| No |  |  |

**D**. How important is this (rest and sleep) to you?

Not at all important, Somewhat unimportant, Neutral, Somewhat important, Very important

**Question 20**

| **A.** Do you need nursing care/support/aids in connection with moving (in or out of bed)/being physically active? | | |
| --- | --- | --- |
| Yes |  |  |
| No |  |  |

| **B.** Have you felt that you received the support/had the aid you needed for this? | | |
| --- | --- | --- |
| Yes |  | If NO: Would you like to describe what you have been missing? |
| No |  |  |

| **C.** Considering your situation, have you had the opportunity to move/be physically active in a way that feels appropriate/good for you? | | |
| --- | --- | --- |
| Yes |  | If NO: What do you consider the main reason for this? |
| No |  |  |

**D**. How important is this (moving/being physically active) to you?

Not at all important, Somewhat unimportant, Neutral, Somewhat important, Very important

**Question 21**

| **A.** Do you need nursing care/support/aids in relation to any physical symptoms you are experiencing (e.g., pain or constipation)? | | |
| --- | --- | --- |
| Yes |  | If YES: What physical symptoms are you experiencing? |
| No |  |  |

Ask questions 21B and 21C for each symptom mentioned:

| **B.** Have you felt that you received nursing care/support to help with these symptoms? | | |
| --- | --- | --- |
| Yes |  | If NO: Would you like to describe what you have been missing? |
| No |  |  |

**C.** How important is this (control of physical symptoms) to you?

Not at all important, Somewhat unimportant, Neutral, Somewhat important, Very important

*Thank you very much for your participation. Is there anything else you would like to ask or add?*
